# Would you choose to be a psychiatrist again? A large-sample nationwide survey of psychiatrists and psychiatry residents in China

**DOI:** 10.1186/s13033-023-00614-6

**Published:** 2023-12-05

**Authors:** Mengyue Gu, Liucheng Zheng, Jingyang Gu, Song Wang, Yudong Shi, Feng Jiang, Huanzhong Liu, Yi-lang Tang

**Affiliations:** 1 Department of Psychiatry, Chaohu Hospital of Anhui Medical University, Hefei, China; 2 Department of Psychiatry, School of Mental Health and Psychological Sciences, Anhui Medical University, Hefei, China; 3 Institute of Healthy Yangtze River Delta, Shanghai Jiao Tong University, Shanghai, China; 4 School of International and Public Affairs, Shanghai Jiao Tong University, Shanghai, China; 5 Department of Psychiatry and Behavioral Sciences, Emory University, Atlanta, Georgia USA; 6 Atlanta VA Medical Center, Decatur, Georgia USA

**Keywords:** Intention of career choice, Psychiatrist, Cross-sectional survey, Job satisfaction, Professional identity

## Abstract

**Background:**

The mental health workforce sustainability in China suffers high rates of attrition and the intention to leave. Among current professionals, the intention to choose the same career is an interesting way to gauge their job satisfaction and other factors, and it may affect the career choices of younger generations. We aimed to survey the intention of psychiatrists and psychiatry residents to choose the same career if they could start over and to identify associated factors.

**Methods:**

We conducted an anonymous survey of psychiatrists in 41 tertiary psychiatric hospitals in China. We collected demographic data, work-related information, the sense of professional identity, job satisfaction, and burnout (Maslach Burnout Inventory), and we specifically asked each participant whether they would choose to be a psychiatrist again if they could.

**Results:**

Among 3,783 psychiatrists we surveyed, one-quarter responded that they would not choose to be a psychiatrist again if they had a choice, with less than half (47.2%) saying they would. Those who would not choose psychiatry again were more likely to have a negative (relative to positive) professional identity (OR = 7.47, P＜0.001, 95%CI: 4.587–12.164); experience job burnout (OR = 2.945, P＜0.001, 95%CI: 2.356–3.681); be dissatisfied with their job (OR = 2.739, P＜0.001, 95%CI: 2.102–3.569) and excessive regulation (OR = 1.819, P＜0.001, 95%CI: 1.487–2.226); have a heavy workload (OR = 1.749, P＜0.001, 95%CI: 1.423–2.149) or a lower income (OR = 1.748, P＜0.001, 95%CI: 1.415–2.161); be married (relative to single) (OR = 1.604, P = 0.004, 95%CI: 1.165–2.208); be dissatisfied with strained doctor-patient relationship (OR = 1.333, P = 0.005, 95%CI: 1.089–1.632); have more night shifts per month (OR = 1.055, P = 0.021, 95%CI: 1.008–1.104) or work longer hours per week (OR = 1.016, P = 0.001, 95%CI: 1.006–1.025).

**Conclusion:**

Among psychiatrists in tertiary hospitals in China, those with a heavier workload, poor sense of professional identity, job dissatisfaction, and burnout were less likely to choose psychiatry again. Policymakers and hospital administrators need to take effective measures to improve psychiatrists’ sense of professional identity and increase their intention to stay.

## Background

China has undergone rapid economic and social transformations in the last 30 years, which have brought about increased psychological stress and various mental issues [1, 2]. According to a study published in 2009, it was estimated that 173 million Chinese adults suffered from mental disorders [3]. The 2019 China Mental Health Survey [4] showed that the lifetime prevalence of mental disorders in China is 16.6% and accounts for the largest proportion (17%) of the global burden of mental, neurological, and substance use disorders [5]. Mental disorders pose a major disease burden to China’s public health system.

However, China’s mental health resources are insufficient and unevenly distributed to meet the increasing demand for mental health services from the public [6]. A survey conducted in 2010 showed that there were only 757 specialized mental health facilities nationwide, with a total of 68,796 healthcare professionals, including 20,480 psychiatrists (1.54 per 100,000) [7]. This means China had less than 2 psychiatrists per 100,000 people in the 2010s, far below the global average. Moreover, most of these health resources are concentrated in the relatively affluent Eastern and Northeastern regions, leaving the rest of the country underserved [7].

Facing growing mental health challenges, China has adopted various measures to enhance the training and development of mental health professionals [8]. However, the number of psychiatrists in China is still insufficient, with less than 3 psychiatrists per 100,000 people according to a more recent survey [9]. The overall ratio of psychiatrists per bed was 0.16 [6], which is far lower than that of developed countries, and even lower than some countries with similar economic levels [10]. This implies that psychiatrists working in China are often burdened with a heavier workload.

Many psychiatrists in China are experiencing burnout, which further contributes to their job dissatisfaction [11, 12]. Studies show that physicians who are satisfied with their jobs are more productive and change jobs less often than those who are dissatisfied [13]. In China, about one-fifth of psychiatrists report an intention to leave their jobs [14]. However, it is unclear whether they would still choose psychiatry as their career if they had another chance. This is an important question because if psychiatrists leave their profession and switch to other fields, it will worsen the shortage of the mental health workforce and jeopardize the quality of mental health services. This may ultimately hamper the reform of the mental health system. Therefore, we need to pay attention to the retention and sustainability of mental health talents.

Psychiatrists’ intention to choose the same career path has been studied in other countries since 2004 [15]. According to an Australian survey of psychiatrists’ job satisfaction and stress levels, respondents were asked whether they would choose to be a psychiatrist again if they could go back in time. The majority (69%) answered in the affirmative. Not wanting to do psychiatry again was independently associated with not being proud (OR = 3.5), not being satisfied (OR = 2.6), and feeling stressed (OR = 1.8). Those who said no were likely to have regretted becoming doctors in general, not just psychiatrists [15]. Similarly, a small proportion (14.1%) of psychiatrists answered “no” to the question in a Finnish national study [16]. Data from a survey of physician salaries in the US last year also showed that 87% of psychiatrists would like to choose to practice psychiatry again [17]. In contrast, the situation in our sample is concerning. A 2018 survey of psychiatrists in 32 psychiatric hospitals across China showed that only 24% of psychiatrists would choose to become a psychiatrist again [18]. This poses a serious challenge for mental health services, as it may cause psychiatrists to leave and discourage new entrants. Therefore, this study aims to explore the factors that influence psychiatrists’ decision to choose psychiatry again and to provide some recommendations for improving the mental health workforce in China.

## Materials and methods

### Design and participants

The National Health Commission of China (NHC) funded the National Hospital Performance Evaluation Survey (NHPES) 2021, which aimed to assess the mental health services resources in tertiary psychiatric hospitals. The survey was part of the NHPES-2021, and it was carried out online from January 11 to March 15, 2021, using the official WeChat account of the NHC, “Healthy China”. Only one response per WeChat account was allowed to avoid duplication. The survey invited 4,899 psychiatrists from 41 hospitals in 28 provinces across China, and 3,973 responses were received, with a response rate of 81.1%. After excluding invalid or incomplete responses, 3,783 responses were included in the analysis. We compared the gender, marital status, and education of the valid and invalid respondents and found no significant differences (t = 0.277, 4.041, and 2.04 respectively, all P > 0.05). The questionnaires were anonymous to protect the participants’ privacy and ensure the results’ validity. The study protocol was approved by the Ethics Committee of the Anhui Medical University Hospital (approval number 202002-kyxm-02). All participants consented to participate in this study.

### Demographic characteristics

Demographic characteristics were collected including age, gender, marital status (married, single, and others), annual income, and educational level (bachelor’s degree or below, master’s degree or above).

### Work-related factors

Work-related factors included professional title (junior or below, mid-level, senior or above), working hours per week, night shifts per month, number of outpatients per week and inpatients per day, participation in the frontline work of COVID-19 (yes or no), dissatisfaction with job-related factors (dissatisfied with income/heavy workload/not feeling respected/strained doctor-patient relationship/excessive regulations) [18–20].

### Professional identity and the decision to be a psychiatrist again

Professional identity was assessed by a single-item question: “Do you identify with your current occupation (negative: not or rarely identified, positive: sometimes or often identified)?”. Consistent with the extant literature [15, 21], participants were asked whether they would choose to become psychiatrists if they could choose again. As an outcome variable, participants responding “no” were categorized as “not wishing to be a psychiatrist again”, “yes” or “uncertain” were categorized as “wishing to be a psychiatrist again or uncertain”.

### Job satisfaction

A short version of the Minnesota Satisfaction Questionnaire (MSQ-SF) was used to assess job satisfaction, which has 20 items [22]. The items are rated on a 5-point Likert-type scale according from 1 = very dissatisfied, to 5 = very satisfied. All item scores are summed to form a general job satisfaction ranging from 20 to 100, with 80 and above indicating “satisfied”, and those below 80 were classified as ‘dissatisfaction’ [23]. The Chinese version of the MSQ-SF has been widely used and proven to offer good stability and validity [24–26]. The Cronbach’s alpha value for this sample in this study was 0.964, indicating a high level of internal consistency.

### Professional burnout

The Maslach Burnout Inventory-Human Service Survey (MBI-HSS) [27] was used to evaluate professional burnout. The scale consists of 22 items, on a 7-point scale from 0 (never) to 6 (every day) depending on the frequency of symptoms, including 3 dimensions: emotional exhaustion (with 9 items: 1, 2, 3, 6, 8, 13, 14, 16, 20), depersonalization (with 5 items: 5, 10, 11, 15, 22) and personal accomplishment (with 8 items: 4, 7, 9, 12, 17, 18, 19, 21). The emotional exhaustion and depersonalization dimensions were scored in the positive direction and the personal accomplishment dimension was scored in the negative direction. Individuals with high emotional exhaustion (≥ 27 points) or depersonalization (≥ 10 points) were considered to be ‘burnout’ [28, 29]. The version of the MBI-HSS in Chinese is from this book [30] which has been widely used and has demonstrated good reliability and validity [30–33]. The scale has a Cronbach’s alpha of 0.833 for the current sample, and Cronbach’s alpha values for the dimensions of emotional exhaustion, depersonalization, and personal accomplishment were 0.912, 0.751, and 0.899 respectively.

### Statistical analysis

All analyses were conducted using the Statistical Package for Social Sciences [34]. Descriptive statistics were performed on demographic characteristics and job-related information. The Kolmogorov-Smirnov test was used to test whether continuous variables were normally distributed. For normally distributed continuous variables, one-way ANOVA was used to test for differences among those who did not wish to be psychiatrists again and those who were or were unsure. For non-normally distributed continuous variables, the Kruskal-Wallis H test was used. A chi-square test was used for categorical variables. Independent variables are significant at *P* < 0.05 and were further analyzed with multiple logistic regression to determine the strength of association between the decision of not wishing to be a psychiatrist again (outcome variable) and other variables.

## Results

### Demographics and work-related information of psychiatrists

As shown in Table 1, the mean age of participants was 38.8 years (SD = 8.7), and they were predominantly female, accounting for 59.8% of the whole sample. Nearly 4/5 were married (79.5%). The median annual income for this group was 120,000 RMB (equivalent to US $1,8461.5). Of the total participants, 1,345 (35.6%) had a master’s degree or above. 36.7% had a senior title and about a quarter had participated in the frontline work of COVID-19 at the time of the survey. The median working hours per week was 48. The median number of daily inpatients and outpatients per week were 15 and 30 respectively. Concerning job dissatisfaction factors, about half participants were dissatisfied with income, heavy workload, and strained doctor-patient relationships.

**Table 1:** Comparison of demographic characteristics, work-related factors, occupational identity, job satisfaction, and burnout between three groups

Variables	Total Sample (n = 3783)	No (n = 961)	Uncertain (n = 1037)	Yes (n = 1785)	*F*/*χ*^2^/*Z*	*p*
Age	38.8 ± 8.7	39.1 ± 7.8	38.1 ± 8.4	39 ± 9.3	4.272	0.014
Gender (%)						
Male	1521(40.2)	444(29.2)	371(24.4)	706(46.4)	23.153	＜ 0.001
Female	2262(59.8)	517(22.9)	666(29.4)	1079(47.7)		
Marital status (%)						
Single	631(16.7)	126(20)	188(29.8)	317(50.2)	14.142	0.007
Married	3008(79.5)	793(26.4)	818(27.2)	1397(46.4)		
Others	144(3.8)	42(29.2)	31(21.5)	71(49.3)		
Annual income (Ten thousand RMBs)^a^	12(9,20)	10(8,15)	12(9,18)	13(10,20)	507.367	＜ 0.001
Educational level (%)						
Bachelor or below	2438(64.4)	681(27.9)	676(27.7)	1081(44.3)	29.286	＜ 0.001
Master or above	1345(35.6)	280(20.8)	361(26.8)	704(52.3)		
Professional title (%)						
Junior or below	1031(27.3)	229(22.2)	305(29.6)	497(48.2)	28.603	＜ 0.001
Mid-level	1364(36.1)	396(29)	388(28.4)	580(42.5)		
Senior or above	1388(36.7)	336(24.2)	344(24.8)	708(51)		
Working hours per week	48(40,56)	50(44,60)	48(40,57)	45(40,54)	4.621	0.032
Night shifts per month	3(1,4)	4(2,5)	3(1,5)	3(0,4)	87.869	＜ 0.001
Number of outpatients per week	30(10,70)	30(10,70)	30(5,70)	30(10,70)	677.708	＜ 0.001
Number of inpatients per day	15(8,20)	15(10,20)	14(7,20)	13(6,20)	21.489	＜ 0.001
Participate in the frontline work of COVID-19 (%)						
Yes	948(25.1)	242(25.5)	260(27.4)	446(47)	2.097	0.718
No	2776(73.4)	705(25.4)	756(27.2)	1315(47.4)		
Dissatisfaction with job-related factors (%)						
Dissatisfied with income	2195(58.0)	710(32.3)	656(29.9)	829(37.8)	209.183	＜ 0.001
Heavy workload	1714(45.3)	616(35.9)	502(29.3)	596(34.8)	243.303	＜ 0.001
Not feeling respected	1216(32.1)	419(34.5)	325(26.7)	472(38.8)	84.737	＜ 0.001
Strained doctor-patient relationship	1891(50.0)	563(29.8)	549(29)	779(41.2)	60.786	＜ 0.001
Excessive regulations	1463(38.7)	539(36.8)	433(29.6)	491(33.6)	220.87	＜ 0.001
Professional identity (%)						
Positive	3555(94.0)	792(22.3)	1000(28.1)	1763(49.6)	310.219	＜ 0.001
Negative	228(6.0)	169(74.1)	37(16.2)	22(9.6)		
Job satisfaction (%)						
Satisfied	968(25.6)	90(9.3)	138(14.3)	740(76.4)	451.028	＜ 0.001
Dissatisfaction	2815(74.4)	871(30.9)	899(31.9)	1045(37.1)		
Burnout(%)	948(25.1)	440(46.4)	288(30.4)	220(23.2)	378.027	＜ 0.001
Emotional exhaustion	11(6,21)	19(11,29)	14(9,22)	8(3,13)	0.198	0.656
Depersonalization	5(2,9)	7(4,12)	6(3,9)	3(1,6)	0.377	0.539
Personal accomplishment	34(24,41)	29(21,37)	32(22,39)	38(29,44)	28.808	＜ 0.001

^a^US dollar to RMB (renminbi) ratio: 1 US dollar ≈ 6.5 RMB.

### Job satisfaction, burnout, occupational identity, and the intention to be a psychiatrist again

**Fig. 1:**
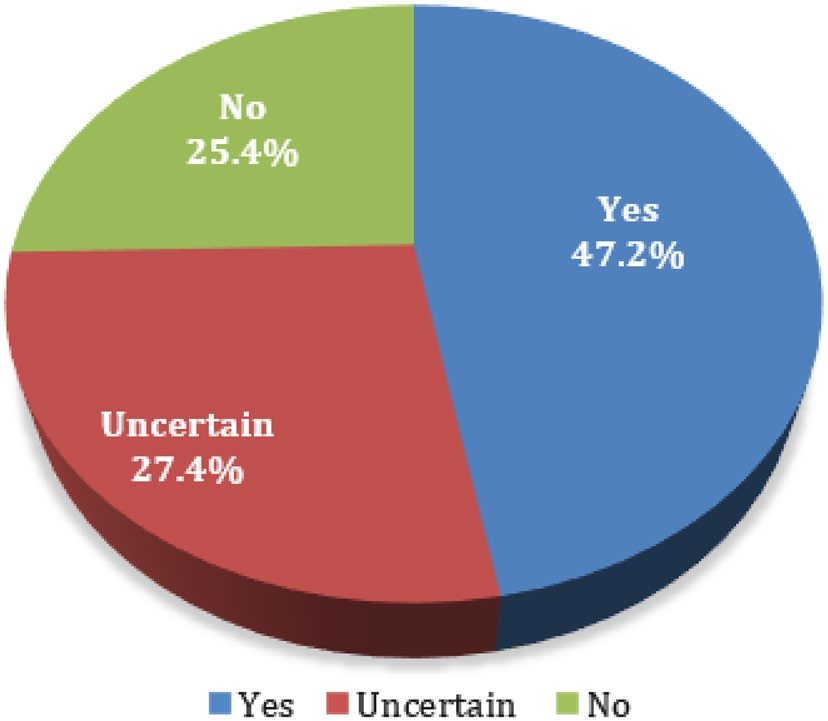
The proportion of psychiatrists and psychiatry residents who were willing to be a psychiatrist again. Participants responding “no” were categorized as “not wishing to be a psychiatrist again”, “yes” or “uncertain” were categorized as “wishing to be a psychiatrist again or uncertain”

In terms of job satisfaction, 968 (25.6%) were satisfied with their current job. The prevalence of burnout was 25.1% (Table 1). 94% had an identification with their occupation. In the decision to be a psychiatrist again, nearly one-quarter of the people (n = 961, 25.4%) in this sample responded negatively while 47.2% responded in the affirmative, and the rest were unsure, as shown in Fig. 1.

### Univariable analysis

In univariate analysis, significant differences were found among those who did not wish to be psychiatrists again and those who were unsure in terms of age, gender, marital status, annual income, educational level, professional title, working hours per week, night shifts per month, number of inpatients per day, the average number of outpatients per week, dissatisfaction with job-related factors, professional identity, job satisfaction, and burnout (all *P* < 0.05). Interestingly, unlike our previous study on psychiatrists’ turnover intention, participation in the frontline work of COVID-19 did not show a significant difference, as shown in Table 1. Furthermore, students graduating from medical school are required to complete residency training before they can begin practicing. During this time, they are referred to as residents. This study included 1,031 psychiatry residents, 27.3% of the total sample. Then, we compared them with other psychiatrists in the decision to choose psychiatry again. It was found that there was a significant difference between the two groups (*F* = 8.433, *P* = 0.015).

### Factors associated with the intention not to be a psychiatrist again

**Fig. 2:**
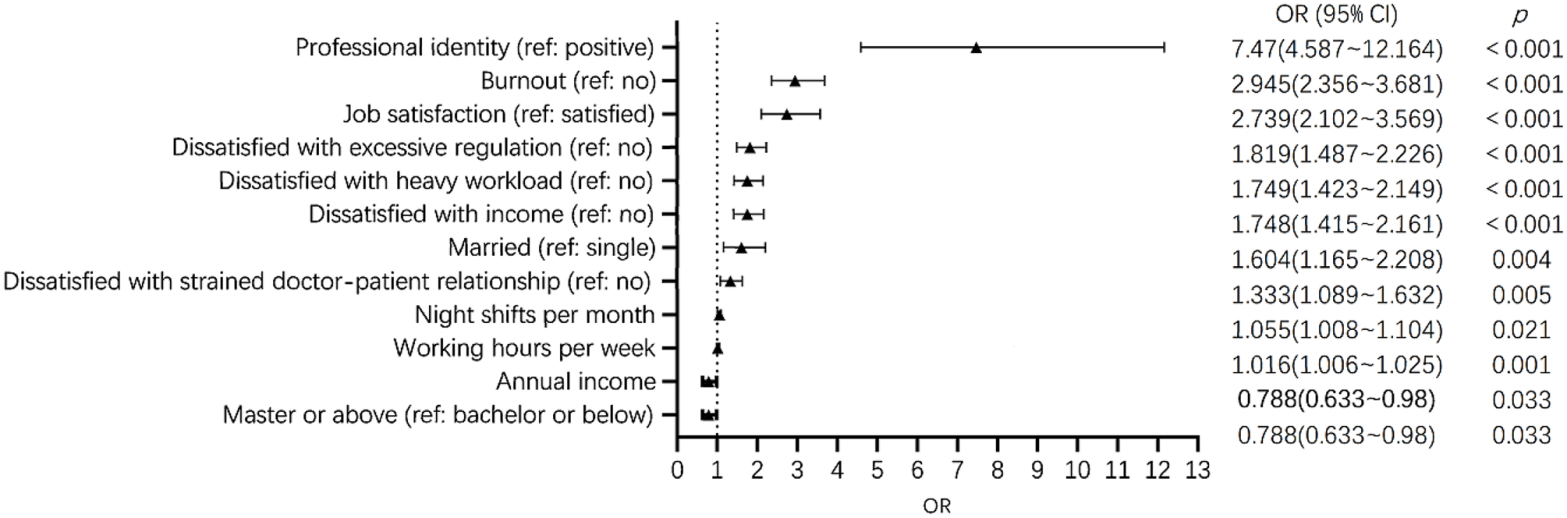
Forest plot for binary logistic regression

Subsequently, we employed a binary logistic regression to examine factors associated with the intention of not wishing to be a psychiatrist again (outcome variable). Collinearity analysis showed that the variance inflation factor (VIF) was less than 3 for all independent variables. As seen in Table 2; Fig. 2, those who would not choose psychiatry again were more likely to have a negative (relative to positive) professional identity (OR = 7.47, P＜0.001, 95%CI: 4.587–12.164); experience job burnout (OR = 2.945, P＜0.001, 95%CI: 2.356–3.681); be dissatisfied with their job (OR = 2.739, P＜0.001, 95%CI: 2.102–3.569) and excessive regulation (OR = 1.819, P＜0.001, 95%CI: 1.487–2.226); have a heavy workload (OR = 1.749, P＜0.001, 95%CI: 1.423–2.149) or a lower income (OR = 1.748, P＜0.001, 95%CI: 1.415–2.161); be married (relative to single) (OR = 1.604, P = 0.004, 95%CI: 1.165–2.208); be dissatisfied with strained doctor-patient relationship (OR = 1.333, P = 0.005, 95%CI: 1.089–1.632); have more night shifts per month (OR = 1.055, P = 0.021, 95%CI: 1.008–1.104) or work longer hours per week (OR = 1.016, P = 0.001, 95%CI: 1.006–1.025).

**Table 2:** Binary logistic regression analysis for the decision to be a psychiatrist again

Variables	B	*p*	OR	95% CI for OR	
				Lower	Upper
Age	0.017	0.07	1.017	0.999	1.035
Female (ref: male)	-0.067	0.51	0.935	0.766	1.141
Marital status					
Married (ref: single)	0.472	0.004	1.604	1.165	2.208
Divorced or other (ref: single)	0.244	0.397	1.276	0.726	2.243
Annual income	-0.239	0.033	0.788	0.633	0.98
Educational level					
Master or above (ref: bachelor or below)	-0.239	0.033	0.788	0.633	0.98
Professional title					
Mid-level (ref: junior or below)	0.18	0.225	1.197	0.895	1.6
Senior or above (ref: junior or below)	-0.039	0.839	0.961	0.656	1.408
Working hours per week	0.016	0.001	1.016	1.006	1.025
Night shifts per month	0.053	0.021	1.055	1.008	1.104
Number of outpatients per week	-0.001	0.065	0.999	0.998	1
Number of inpatients per day	-0.004	0.199	0.996	0.989	1.002
Dissatisfaction with job-related factors					
Dissatisfied with income (ref: no)	0.559	＜0.001	1.748	1.415	2.161
Heavy workload (ref: no)	0.559	＜0.001	1.749	1.423	2.149
Not feeling respected (ref: no)	0.127	0.251	1.135	0.914	1.409
Strained doctor-patient relationship (ref: no)	0.287	0.005	1.333	1.089	1.632
Excessive regulation (ref: no)	0.598	＜0.001	1.819	1.487	2.226
Professional identity (ref: positive)	2.011	＜0.001	7.47	4.587	12.164
Job satisfaction (ref: satisfied)	1.008	＜0.001	2.739	2.102	3.569
Burnout (ref: no)	1.08	＜0.001	2.945	2.356	3.681
Constant	-4.343	＜0.001	0.013		

VIF was less than 3 for all independent variables; R^2^ = 0.402

## Discussion

To the best of our knowledge, this is the first national survey focusing on psychiatrists’ intention to choose to be a psychiatrist again if they had another choice and the associated factors. Unlike previous studies that focused on psychiatrists’ turnover intention and job satisfaction, this study focused on physicians’ intention to choose psychiatry again by asking a hypothetical question. The survey was conducted among psychiatrists from 41 tertiary psychiatric hospitals across China and yielded mixed results. About a quarter (25.4%) of psychiatrists answered they would not choose to become a psychiatrist again, and their decision was associated with factors such as marital status (married), lower annual income, longer working hours, and job dissatisfaction. Our findings offer possible preventive strategies that may help reduce the attrition of psychiatrists from the field and enhance their retention and identification with the profession.

Mental health is an important public health and social issue that affects economic and social development, but it faces great challenges in China. The National Mental Health Work Plan (2015–2020) aimed to improve the capacity of mental health services, implement national policies on salaries and benefits of mental health staff, improve their remuneration levels, and stabilize the workforce of mental health professionals. This study found two positive outcomes of these measures. Compared to a 2018 survey where only 24% of psychiatrists would choose the same career again [21], our study showed 47.2% answered yes and this is encouraging. Moreover, our survey found that the number of highly educated psychiatrists has increased nearly sixfold compared to 10 years ago, with 35.6% of psychiatrists having a master’s or doctoral degree in medicine, which means that the quality of the psychiatric workforce has improved significantly [7]. However, this may be influenced by the sampling effect, as our study was conducted in top-tier (tertiary) hospitals, where the best local health resources are concentrated and doctors are better educated and trained [6, 35]. Nevertheless, there are still areas that need improvements and changes. Only about a quarter of psychiatrists were satisfied with their work, and more than half were reluctant or unsure about becoming psychiatrists again. This is still a large gap compared to the data of foreign countries [15–17].

Interestingly, our study found that among demographic characteristics, marital status was associated with psychiatrists’ intention to choose psychiatry again. Compared to single, married psychiatrists were less likely to choose psychiatry again. This may be because married psychiatrists are required to take on more family responsibilities. The role of a physician makes it difficult to maintain a work-life balance, thus creating conflicts and making psychiatrists regret their initial career choice. Here it is more likely that they regret their choice to become a doctor in general rather than a psychiatrist.

We found that psychiatrists with lower annual incomes, more night shifts per month, and longer working hours per week were less likely to choose psychiatry again. Previous studies have shown that salary is a crucial factor affecting physicians’ job satisfaction [36], and low pay may lead to job dissatisfaction [37]. Although it is unclear that job dissatisfaction led physicians to leave their profession, some studies have found that job satisfaction was associated with turnover intentions among physicians in general [38, 39] and psychiatrists in particular [14]. This sample’s average working hours per week was 48 h, much higher than the legal limit of 40 h in China. Overwork has been shown to contribute to job burnout among doctors [40, 41], and doctors in China are not paid in proportion to their working hours [36]. Therefore, increasing physicians’ income and reducing their working hours are possible solutions. This was also reflected in the dissatisfaction with job-related factors. Psychiatrists who were dissatisfied with their income and heavy workload were less likely to choose psychiatry again. This aligns with previous findings that doctors often complained about low salaries and heavy workloads [38, 39]. A survey in Zhejiang Province [19] found that 88% of doctors did not want their children to follow in their footsteps, citing low pay and heavy workload as the two main reasons. One reason psychiatrists would not choose their specialty again was their dissatisfaction with excessive regulations. To prevent or reduce burnout among doctors, it is essential to eliminate or reduce factors in the work environment that do not enhance the sense of meaning in their work, such as excessive regulation and paperwork [42–44].

We confirmed that job dissatisfaction and burnout were two strong predictors that contributed to psychiatrists’ regret in choosing their profession [14, 45]. Previous studies have found that physicians with higher job satisfaction tend to be more enthusiastic about their work and get more accomplished from the work, which in turn reduces the incidence of burnout [45]. However, this study also revealed a paradoxical result: despite a lower rate (25.1%) of burnout, a rate that was significantly lower than 38.4% in 2019 [37], only 25.6% of psychiatrists were satisfied with their jobs, which was also a decline from a previous surveying a similar sample [37]. This discrepancy may be due to the different criteria used to measure job satisfaction in this study, which were more stringent than in previous ones. Job satisfaction is a complex construct that reflects an individual’s emotional response and behavioral expression to their work, work environment, and work life, and is more specific to their current job [46]. Therefore, this study suggests that improving job satisfaction and reducing burnout are key strategies to retain psychiatric talents, especially at the hospital level where staff support and wellness programs should be developed and accessible to psychiatrists [47].

Previous research among general practitioners did not find a direct link between professional identity and turnover intention [45]. However, our study found that professional identity was the strongest predictor (OR = 5.486) of the intention to be a psychiatrist again. Professional identity refers to an individual’s perception of the social significance and social value of the profession in which they are engaged, and it has a positive impact on an individual’s self-image, professional satisfaction, sense of belonging, and recognition of professional competence [46]. In this study, we focused on how psychiatrists valued the healthcare work they did, rather than their current position. When an individual has a positive sense of identity with their profession, they will be more motivated and passionate about it and will be less affected by negative aspects such as the working environment [48]. Moreover, professional identity can lower the risk of burnout [12]. Few found that psychiatrists had a high level of professional identity (94%), which is consistent with findings in other health workers [46, 49]. Therefore, we suggest that enhancing the social recognition and appreciation of the psychiatric profession, as well as awareness of the importance of mental health services, may help retain mental health professionals by strengthening their professional identity.

Several limitations to the study need to be acknowledged. First, we asked participants to respond to a hypothetical scenario about choosing psychiatry as a career, rather than examining the actual outcomes of psychiatrists who left the field. Therefore, we do not know if psychiatrists stay or exit from psychiatry after quitting or retiring. This is an interesting area that needs further research. Our findings should be interpreted with caution. We plan to follow up on the career decisions of departing psychiatrists, observe their alternatives, and identify possible influencing factors in future studies. Second, the study was conducted among psychiatrists working in tertiary psychiatric hospitals, and the findings may not be generalizable to psychiatrists working in other settings, such as rural or remote areas. Third, the study was a cross-sectional survey, and the synchronicity of the measurements of the independent and dependent variables did not allow causal inferences to be drawn between the factors and the outcome variables.

## Conclusions

Based on this nationally representative survey of a large sample of psychiatrists working in tertiary psychiatric hospitals, we found that one-quarter of psychiatrists would not choose to be a psychiatrist if they had a chance to start over. The factors associated with the reluctance to choose psychiatry as their career included lower annual income, poor professional identity, job dissatisfaction, and burnout. Policymakers and hospital administrators need to focus on the identified groups and work-related factors and take effective measures to increase workforce sustainability for current psychiatrists.

Possible measures include increasing physicians’ incomes, reducing workload, streamlining workflow, and improving the doctor-patient relationship. Moreover, they can develop staff care and wellness programs at the hospital level to increase psychiatrists’ job satisfaction, reduce the incidence of burnout, and retain the psychiatric workforce.

## Data Availability

The dataset presented in this article is not readily available due to privacy concerns. Requests to access the datasets should be directed to the corresponding author at fengjiang@sjtu.edu.cn.

## List of abbreviations

MSQ-SF: the Minnesota Satisfaction Questionnaire
MBI-HSS: the Maslach Burnout Inventory-Human Service Survey

## References

[1] Huang Y, Liu Z, Wang H, Guan X, Chen H, Ma C (2016). The China Mental Health Survey (CMHS): I. background, aims and measures. Soc Psychiatry Psychiatr Epidemiol.

[2] Shen YC, Zhang MY, Huang YQ, He YL, Liu ZR, Cheng H (2006). Twelve-month prevalence, severity, and unmet need for treatment of mental disorders in metropolitan China. Psychol Med.

[3] Phillips MR, Zhang J, Shi Q, Song Z, Ding Z, Pang S (2009). Prevalence, treatment, and associated disability of mental disorders in four provinces in China during 2001-05: an epidemiological survey. Lancet.

[4] Huang Y, Wang Y, Wang H, Liu Z, Yu X, Yan J (2019). Prevalence of mental disorders in China: a cross-sectional epidemiological study. Lancet Psychiatry.

[5] Charlson FJ, Baxter AJ, Cheng HG, Shidhaye R, Whiteford HA (2016). The burden of mental, neurological, and substance use disorders in China and India: a systematic analysis of community representative epidemiological studies. Lancet.

[6] Xia L, Jiang F, Rakofsky J, Zhang Y, Shi Y, Zhang K (2021). Resources and workforce in Top-Tier Psychiatric hospitals in China: a Nationwide Survey. Front Psychiatry.

[7] Liu C, Chen L, Xie B, Yan J, Jin T, Wu Z (2013). Number and characteristics of medical professionals working in Chinese mental health facilities. Shanghai Arch Psychiatry.

[8] Xiong W, Phillips MR (2016). Translated and annotated version of the 2015–2020 National Mental Health Work Plan of the people’s Republic of China. Shanghai Arch Psychiatry.

[9] Shi CMN, Wang L, Yi L, Wang X, Zhang W (2019). Study of the mental health resources in China. Chin J Health Policy.

[10] WHO, Mental Health ATLAS. 2017. Geneva: World Health Organization 2018 [.

[11] Baumgardt J, Moock W Jr, Kawohl W. Aspects of sustainability: cooperation, job satisfaction, and burnout among Swiss psychiatrists. Front Public Health. 2015;3. 10.3389/fpubh.2015.00025. 10.3389/fpubh.2015.00025 PMC4323987 25717469

[12] Scanlan JN, Still M (2019). Relationships between burnout, turnover intention, job satisfaction, job demands and job resources for mental health personnel in an Australian mental health service. BMC Health Serv Res.

[13] Haas JS (2001). Physician discontent: a barometer of change and need for intervention. J Gen Intern Med.

[14] Yang Y, Zhang L, Li M, Wu X, Xia L, Liu DY, et al. Turnover intention and its associated factors among psychiatrists in 41 tertiary hospitals in China during the COVID-19 pandemic. Front Psychol. 2022;13. 10.3389/fpsyg.2022.899358. 10.3389/fpsyg.2022.899358 PMC9226451 35756286

[15] Rey JM, Walter G, Giuffrida M (2004). Australian psychiatrists today: proud of their profession but stressed and apprehensive about the future. Aust N Z J Psychiatry.

[16] Heikkilä TJ, Hyppölä H, Vänskä J, Halila H, Kujala S, Virjo I (2016). What predicts doctors’ satisfaction with their chosen medical specialty? A Finnish national study. BMC Med Educ.

[17] Leslie Kane M, Medscape Physician CR. 2022: Incomes gain, pay gaps remain 2022 [Available from: https://www.medscape.com/slideshow/2022-compensation-overview-6015043?icd=login_success_email_match_norm#20.

[18] Jiang F, Zhou H, Rakofsky J, Hu L, Liu T, Wu S (2019). Intention to leave and associated factors among psychiatric nurses in China: a nationwide cross-sectional study. Int J Nurs Stud.

[19] Wu D, Wang Y, Lam KF, Hesketh T (2014). Health system reforms, Violence against doctors and job satisfaction in the medical profession: a cross-sectional survey in Zhejiang Province, Eastern China. BMJ Open.

[20] Zhang L, Li M, Yang Y, Xia L, Min K, Liu T (2022). Gender differences in the experience of burnout and its correlates among Chinese psychiatric nurses during the COVID-19 pandemic: a large-sample nationwide survey. Int J Mental Health Nurs.

[21] Jiang F, Hu L, Rakofsky J, Liu T, Wu S, Zhao P (2018). Sociodemographic Characteristics and job satisfaction of psychiatrists in China: results from the First Nationwide Survey. Psychiatr Serv.

[22] Weiss DJ, Dawis RV, England GW. Manual for the Minnesota Satisfaction Questionnaire. Minnesota Studies in Vocational Rehabilitation. 1967.

[23] Sharp TP (2008). Job satisfaction among psychiatric registered nurses in New England. J Psychiatr Ment Health Nurs.

[24] Liu D, Wu Y, Jiang F, Wang M, Liu Y, Tang YL (2021). Gender differences in job satisfaction and work-Life Balance among Chinese Physicians in Tertiary Public hospitals. Front Public Health.

[25] Lu Y, Hu XM, Huang XL, Zhuang XD, Guo P, Feng LF (2016). Job satisfaction and associated factors among healthcare staff: a cross-sectional study in Guangdong Province, China. BMJ Open.

[26] Zhou H, Jiang F, Rakofsky J, Hu L, Liu T, Wu S (2019). Job satisfaction and associated factors among psychiatric nurses in tertiary psychiatric hospitals: results from a nationwide cross-sectional study. J Adv Nursing.

[27] Maslach CJS, Leiter MP. Maslach burnout inventory: third edition.In: Evaluating stress: a book of resources. Lanham, MD, US: Scarecrow Education. 1997:191–218.

[28] Rafferty JP, Lemkau JP, Purdy RR, Rudisill JR (1986). Validity of the Maslach Burnout Inventory for family practice physicians. J Clin Psychol.

[29] Thomas NK (2004). Resident burnout. JAMA.

[30] Dail L et al. Fields (US), translated by Yang Zhiping. Taking the measure of work: A guide to validated scales for organizational research and diagnosis: Beijing: China Light Industry Press; 2004. 271 p.

[31] Li H, Zuo M, Gelb AW, Zhang B, Zhao X, Yao D (2018). Chinese anesthesiologists have high burnout and low job satisfaction: a cross-sectional survey. Anesth Analg.

[32] Ma S, Huang Y, Yang Y, Ma Y, Zhou T, Zhao H (2019). Prevalence of Burnout and Career satisfaction among oncologists in China: A National Survey. Oncologist.

[33] Zheng H, Shao H, Zhou Y (2018). Burnout among Chinese adult reconstructive surgeons: incidence, risk factors, and Relationship with Intraoperative Irritability. J Arthroplasty.

[34] SPSS. IBM SPSS statistics for Windows, Version 23.0. Armonk, NY: IBM Corp. version 23.

[35] Zhu J, Li W, Chen L (2016). Doctors in China: improving quality through modernisation of residency education. Lancet.

[36] Zhang C, Liu Y (2018). The salary of physicians in Chinese public tertiary hospitals: a national cross-sectional and follow-up study. BMC Health Serv Res.

[37] Yao H, Wang P, Tang YL, Liu Y, Liu T, Liu H (2021). Burnout and job satisfaction of psychiatrists in China: a nationwide survey. BMC Psychiatry.

[38] Wang Z, Xie Z, Dai J, Zhang L, Huang Y, Chen B (2014). Physician burnout and its associated factors: a cross-sectional study in Shanghai. J Occup Health.

[39] Zhang Y, Feng X (2011). The relationship between job satisfaction, burnout, and turnover intention among physicians from urban state-owned medical institutions in Hubei, China: a cross-sectional study. BMC Health Serv Res.

[40] Dyrbye LN, Varkey P, Boone SL, Satele DV, Sloan JA, Shanafelt TD. Physician satisfaction and burnout at different career stages. Mayo Clin Proc. 2013;88(12):1358-67. 10.1016/j.mayocp.2013.07.016. 10.1016/j.mayocp.2013.07.016 24290109

[41] Hoff T, Carabetta S, Collinson GE (2019). Satisfaction, Burnout, and turnover among nurse practitioners and physician assistants: a review of the empirical literature. Med Care Res Rev.

[42] Dyrbye LN, West CP, Burriss TC, Shanafelt TD (2012). Providing primary care in the United States: the work no one sees. Arch Intern Med.

[43] Shanafelt TD, Dyrbye LN, Sinsky C, Hasan O, Satele D, Sloan J et al. Relationship Between Clerical Burden and Characteristics of the Electronic Environment With Physician Burnout and Professional Satisfaction. Mayo Clin Proc. 2016;91(7):836 – 48. 10.1016/j.mayocp.2016.05.007. 10.1016/j.mayocp.2016.05.007 27313121

[44] Sinsky C, Colligan L, Li L, Prgomet M, Reynolds S, Goeders L (2016). Allocation of Physician Time in Ambulatory practice: a Time and Motion Study in 4 specialties. Ann Intern Med.

[45] Zhang T, Feng J, Jiang H, Shen X, Pu B, Gan Y (2021). Association of professional identity, job satisfaction and burnout with turnover intention among general practitioners in China: evidence from a national survey. BMC Health Serv Res.

[46] Sabanciogullari S, Dogan S (2015). Relationship between job satisfaction, professional identity and intention to leave the profession among nurses in Turkey. J Nurs Manage.

[47] West CP, Dyrbye LN, Erwin PJ, Shanafelt TD (2016). Interventions to prevent and reduce physician burnout: a systematic review and meta-analysis. Lancet.

[48] Coetzee M, van Dyk J (2018). Workplace bullying and turnover intention: exploring Work Engagement as a potential mediator. Psychol Rep.

[49] Li L, Gan Y, Yang Y, Jiang H, Lu K, Zhou X (2020). Analysis on professional identity and related factors among Chinese general practitioners: a National cross-sectional study. BMC Fam Pract.

